# Postoperative Thrombocytopenia after Revision Arthroplasty: Features, Diagnostic and Therapeutic Considerations

**DOI:** 10.3390/life14091124

**Published:** 2024-09-06

**Authors:** Carmen Saguna, Nicoleta Mariana Berbec, Marian Platon, Alexandra Marcoci, Andreea Jercan, Andrei Colita, Mihai Emanuel Gherghe, Dana-Georgiana Nedelea, Romica Cergan, Cristian Scheau, Serban Dragosloveanu

**Affiliations:** 1 Hematology Clinic, Coltea Clinical Hospital, 030171 Bucharest, Romania; 2 Department of Hematology, The “Carol Davila” University of Medicine and Pharmacy, 050474 Bucharest, Romania; 3 Hematology Clinic, “Dr. Carol Davila” Military Emergency Hospital, 010825 Bucharest, Romania; 4 Department of Orthopaedics, “Foisor” Clinical Hospital of Orthopaedics, Traumatology and Osteoarticular TB, 021382 Bucharest, Romania; 5 Department of Anatomy, The “Carol Davila” University of Medicine and Pharmacy, 050474 Bucharest, Romania; 6 Department of Radiology and Medical Imaging, “Foisor” Clinical Hospital of Orthopaedics, Traumatology and Osteoarticular TB, 021382 Bucharest, Romania; 7 Department of Physiology, The “Carol Davila” University of Medicine and Pharmacy, 050474 Bucharest, Romania; 8 Department of Orthopaedics and Traumatology, The “Carol Davila” University of Medicine and Pharmacy, 050474 Bucharest, Romania

**Keywords:** postoperative thrombocytopenia, immune thrombocytopenia, platelets, disseminated intravascular coagulation, immune mechanism, drug-induced, pathophysiology, orthopedics

## Abstract

We present the case of a 66 year-old male patient who developed severe postoperative thrombocytopenia after revision total hip arthroplasty. The patient underwent surgery in a dedicated orthopedics hospital and was initially managed in the intensive care unit. Upon the development of thrombocytopenia, he was referred to a dedicated hematology clinic for investigation and advanced management. A thorough diagnostic algorithm was employed in order to rule out the main causes of thrombocytopenia. By exclusion, we diagnosed the patient as suffering from a rare and severe form of postoperative thrombocytopenia through an immune mechanism. Although postoperative thrombocytopenia is relatively frequent but transitory and no treatment is required, this condition was refractory to corticosteroids and substitution therapy; however, it quickly responded to treatment with thrombopoietin receptor agonists. The patient recovered and was successfully discharged with normal platelet values. While rare occurrences, alternative causes of thrombocytopenia such as infection, drug-induced, or immune should be considered in patients developing postoperative thrombocytopenia.

## 1. Introduction

Thrombocytopenia is defined as a platelet concentration lower than 150 × 10^9^/L and may be encountered after major surgeries in up to 60% of patients [1,2,3]. Concerning patients undergoing orthopedic surgery, the risk of postoperative thrombocytopenia is higher in patients aged > 65 years, yet no significant difference is mentioned in the available literature regarding gender or types of procedure.

It is known that platelet count reduction appears more often in patients with ages above 65 years, compared to those ≤65 years, suggesting that advanced age increases the risk of thrombocytopenia and drawing attention to the importance of close monitoring [3,4]. Early-onset postoperative thrombocytopenia is physiological because surgery elicits complement activation, neutrophil degranulation, and the release of proinflammatory cytokines that cause endothelial cell dysfunction, the release of tissue thromboplastin (tissue factor), and von Willibrand factor, leading to platelet activation, aggregation, and consumption. In most cases, physiologic postoperative thrombocytopenia is mild, with platelet values above 100 × 10^9^/L, is not associated with bleeding, and resolves spontaneously within 3–4 days. If thrombocytopenia persists for more than five days or if the onset is late (more than 5 days), other causes of thrombocytopenia should be considered [3,4].

Arthroplasty is a routine elective surgical procedure in orthopedics [5]. Revision total hip arthroplasty (THA) is performed when the hip implant fails, which can occur due to a variety of factors including infection, loosening, or trauma [6,7,8,9]. The number of primary THA procedures has increased significantly, which has led to an increase in the number of revision THA procedures [10].

We present the case of a patient diagnosed with severe thrombocytopenia after a revision THA who underwent treatment in April 2024. The patient benefited from treatment in two hospitals, and the onset, management, and outcome of the postoperative thrombocytopenia are presented in the following.

## 2. Case Presentation

**Figure 1:**
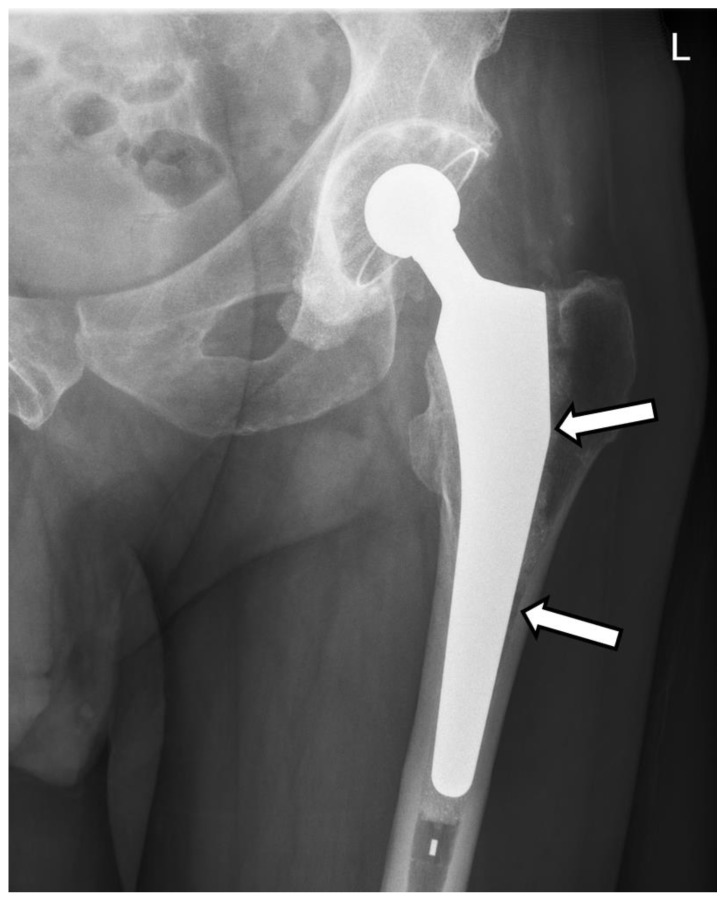
Antero-posterior X-ray of the pelvic ring and a side view of the left hip. Radiological signs of loosening of the femoral component (arrows).

A 66 year-old male patient was referred to the ambulatory department of the “Foisor” Clinical Hospital of Orthopaedics, Traumatology and Osteoarticular TB complaining of persistent left hip pain that started 2 months prior, unrelated to any trauma. The pain increased in intensity, causing functional impairment, and the patient found relief by sitting in a specific position. He had undergone left total hip arthroplasty at the age of 60 due to osteoarthritis. The patient’s medical history included diabetes mellitus type 2 under treatment with Metformin, high blood pressure, a previous complicated gastric ulcer, and iron deficiency. During the clinical assessment, the patient reported experiencing pain in the left groin and thigh when touched or moved gently. He was unable to walk but did not have any other complaints. No vascular or nervous injuries were found, and the skin appeared normal. The anteroposterior X-ray of the pelvic ring and a side view of the left hip showed a loosening of the femoral stem (Figure 1).

### 2.1. Surgical Approach and Management

The patient underwent general anesthesia in a dorsal decubitus position. He received vancomycin 1 g and ceftriaxone 2 g intravenously as antibiotic prophylaxis. A lateral approach (Hardinge) was used to access the left hip, and the fascia lata was opened. Upon exploration, it was confirmed that the fixation was compromised for both the acetabular and femoral components. Samples were taken from the femur and acetabular components for microbiological analysis. The removed material was sent to sonication for microbiological studies.

A cementless acetabular cup (Trilogy^®^ acetabular system, Zimmer Biomet, Warsaw, IN, USA) was considered appropriate and was applied with two screws, along with a significant additional bone graft. A femoral stem (Taperloc femoral system, Zimmer Biomet, Warsaw, IN, USA) was used for femoral revision. Mobility and stability controls were satisfactory. Wash out, hemostasis control, and final closure were performed with one drain.

**Figure 2:**
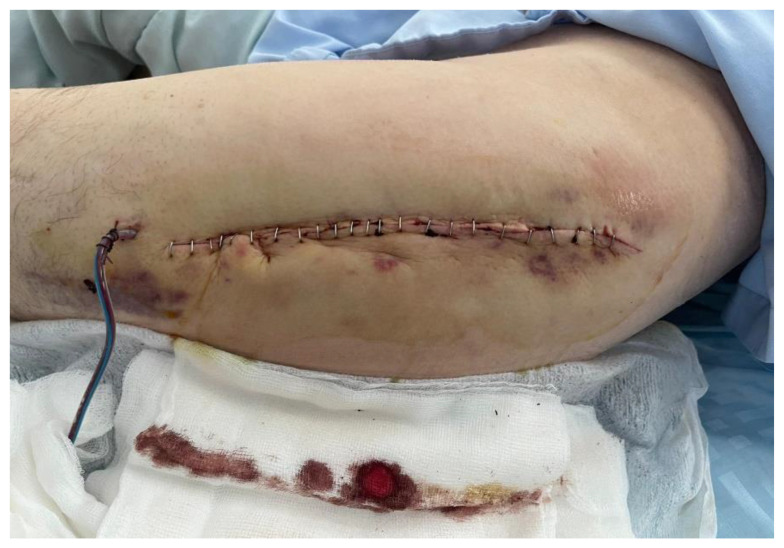
Surgical wound site (drainage tube and staples).

The patient presented a smooth recovery without any complications. The drain was removed on the 3rd day after the surgery, and the staples were removed on the 21st day (see Figure 2). All bacterial samples, including the implant sonication, remained negative after 5 days.

At 6 weeks postoperatively, the patient reported left hip pain when sitting and when performing external rotation during the clinical assessment. Radiographs confirmed the acetabular component malposition.

### 2.2. Surgical Technique and Management of Acetabular Component Revision

**Figure 3:**
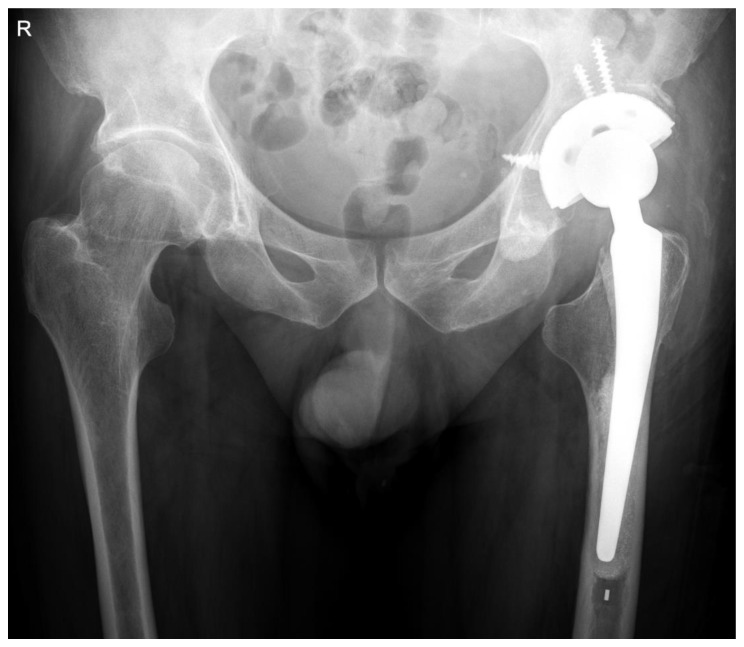
Postoperative radiographical aspect of the revision components.

The approach of the revision surgery was performed through the original incision site, and the screws were removed. However, the left cup was firmly fixed to the acetabular bone and was difficult to remove by hand; therefore, a chisel was inserted between the acetabulum and the cup. After removal, we noted small amounts of bone-like tissue on the posterior surface of the cup and in a screw hole. Cup revision was performed using a titanium revision shell cup (Zimmer Biomet, Warsaw, IN, USA). There were no visible signs of loosening of the stem or infection of the implant. Postoperative radiographs showed a satisfactory outcome (Figure 3).

The intraoperative evolution was marked by complications (massive, oozing bleeding), with post-hemorrhagic anemia—Haemoglobin (Hb) = 8.6 g/dL. The patient did not receive preoperative anticoagulant therapy. Immediate platelet counts showed a significant postoperative decline (2 × 10^9^/L); therefore, the patient received blood products (packed red blood cells and platelet concentrate, ABO and Rh system compatible) and Dexamethasone.

**Figure 4:**
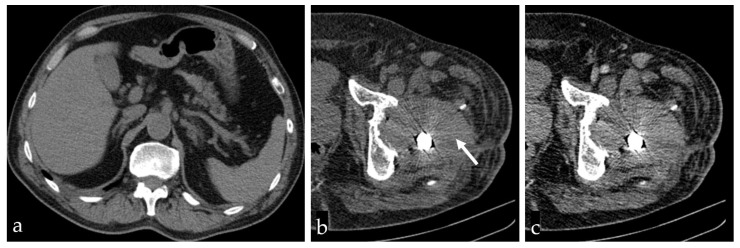
Computed Tomography scan showing normal size of spleen (**a**), recent periprosthetic hematoma with densities of approximately 60–80 HU (**b**, arrow), and no active bleeding, i.e., no pathological enhancement with contrast injection (**c**).

A contrast-enhanced Computed Tomography (CT) scan was performed, showing a recent periprosthetic hematoma with no active bleeding, normally positioned drain tubes, and unremarkable abdominal organs (Figure 4).

The postoperative evolution was stable and favorable, and the patient was monitored in the intensive care unit of Foisor Hospital. Low molecular weight heparin (Clexane) 0.4 mL was administered at 12 h postoperatively in a single dose and was subsequently interrupted as a result of severe thrombocytopenia. However, on postoperative day 1 (POD1), following routine investigations, the patient showed severe thrombocytopenia (grade IV) with a platelet count of 1 × 10^9^/L confirmed on 2 different samples. It was decided to administer Haemocomplettan (fibrinogen concentrate) 1 g, Beriplex (coagulation factor concentrate) 500 U, and corticosteroid therapy (Methylprednisolone), and the transfer to the Hematology Clinic of Coltea Hospital was arranged for hematological evaluation and further investigations.

It should be noted that, before surgery, the patient had mild hypochromic anemia (Hb = 11.2 g/dL, Mean corpuscular hemoglobin concentration (MCHC) = 26.4 g/dL), normal values of platelets (428 × 10^9^/L) and leukocytes (6450/mm^3^). The performed coagulation tests excluded hemostasis disorders (Protrombin time (PT) = 11.3 s, International Normalized Ratio (INR) = 1.94, Activated partial thrombopalstin time (APTT) = 22.8 s, Fibrinogen = 372 mg/dL).

### 2.3. Hematological Evaluation and Investigation

**Figure 5:**
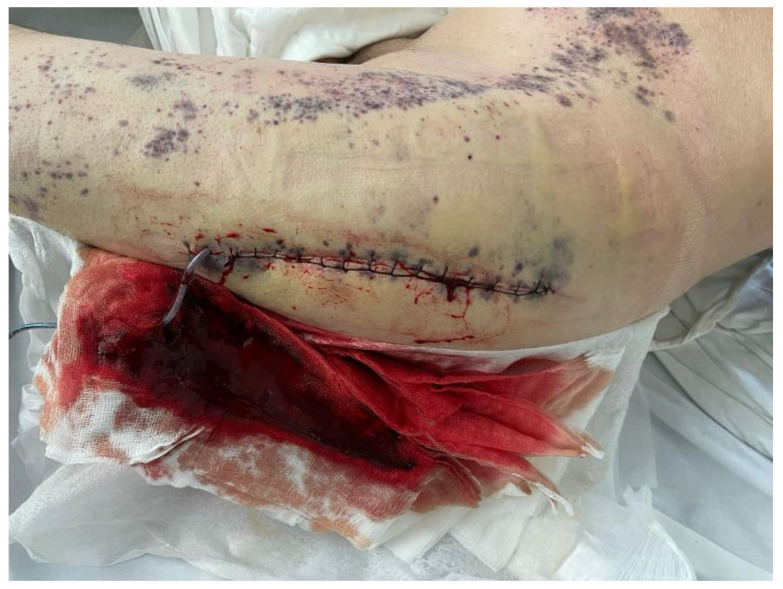
Postoperative wound aspect after the second revision.

Upon admission to the Hematology Clinic, the patient presented with a mediocre general condition, mucocutaneous pallor, petechial and ecchymotic purpura disseminated on the limbs and trunk, hemorrhagic bullae on the jugal mucosa, and gum bleeding; the surgery wound site carried a drainage tube with hemorrhagic liquid (Figure 5); no palpable peripheral adenopathy or hepatosplenomegaly was noticed.

**Figure 6:**
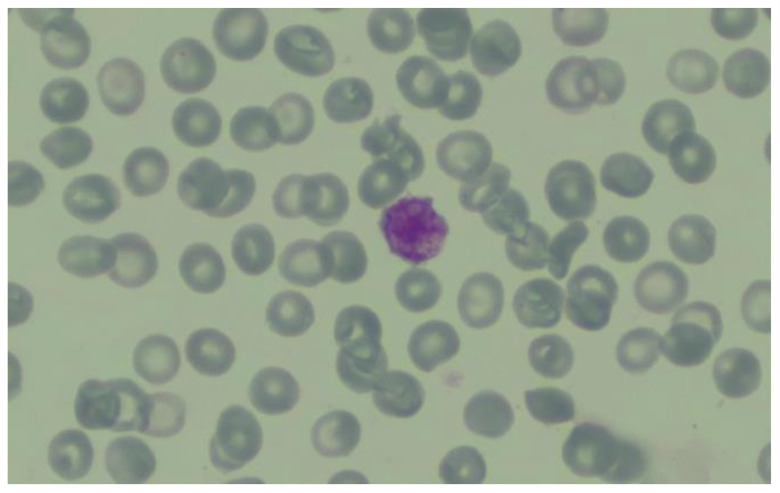
Capillary blood smear—a rare, isolated giant platelet. Courtesy of the Hematology Laboratory of Coltea Hematology Clinic, Bucharest, Romania.

Peripheral blood samples were collected on citrate vials to exclude a possible pseudothrombocytopenia induced by EDTA (unlikely in this case, considering the presence of extensive hemorrhagic syndrome) but also for the correct assessment of the number of platelets. Results showed a moderate microcytic hypochromic anemia of the normoregenerative type (Hb = 7.8 g/dL, Mean cell volume (MCV) =76.2 fL, Mean cell hemoglobin (MCH) = 25.7 pg, MCHC = 33.8 g/dL, reticulocytes = 1.5%) and severe thrombocytopenia (platelet count 7 × 10^9^/L). Examination of the capillary blood smear revealed erythrocyte anisocytosis (microcytosis, hypochromia, rare annulocytes), and the presence of rare isolated platelets altogether with the presence of rare macroplatelets (Figure 6).

**Figure 7:**
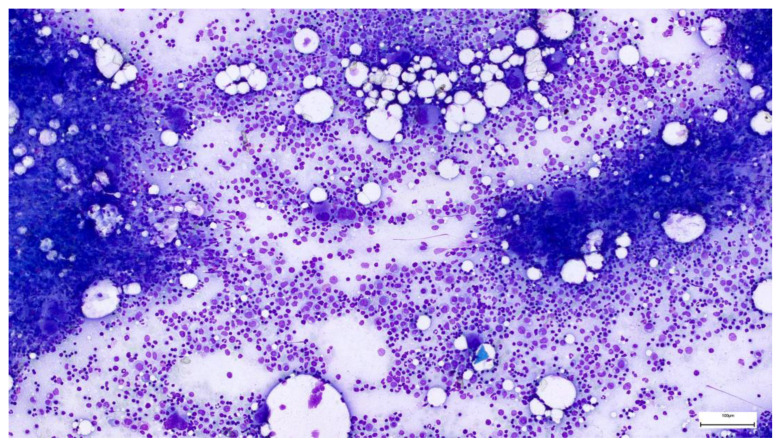
Bone marrow aspirate—megakaryocytic lineage hyperplasia. Courtesy of Hematology Laboratory of Coltea Hematology Clinic, Bucharest, Romania.

We considered that the thrombocytopenia could have theoretically been caused by a pre-existing condition of the bone marrow; however, the morphological examination of the marrow aspirate revealed hypercellular bone marrow, without atypia of the erythroid, granulocytic, and lymphoplasmacytic series. The abundant megakaryocytic series and the presence of precursor elements in all stages of maturation ruled out a central cause through insufficient production of platelets (Figure 7).

We further assessed whether the thrombocytopenia might have had an infectious cause, as in the postoperative phase, infection is not an uncommon complication and both viral and bacterial infections can cause thrombocytopenia [11,12]. The mechanisms responsible could be represented by direct bacteria-platelet interaction followed by immune-mediated destruction or hemophagocytosis of platelets in response to infection. Postoperative thrombocytopenia can also be caused by platelet consumption during sepsis (almost 64% of patients with sepsis), disseminated intravascular coagulation (DIC), or thrombotic microangiopathy (TMA) [12,13]. Of important notice, the patient showed positive serology for chronic hepatitis B and negative serology for hepatitis C and HIV; also, the stool Helicobacter pylori antigen was negative.

The laboratory work-up excluded consumption thrombocytopenia within the DIC. Our analysis showed the absence of fragmented erythrocytes on the capillary blood smear and no significantly prolonged clotting times. The increased value of D-dimers (3.70 [normal range: 0–0.50 µg/mL]) was considered physiological and expected in the postoperative context [14]. Although the patient presented elevated C-reactive protein values, sepsis was ruled out, as the blood cultures and bacteriological swabs were negative; moreover, the procalcitonin and presepsin values were within the normal reference range.

The persistence of bleeding from the surgical wound site and the intensity of the hemorrhagic syndrome required the consideration of primary fibrinolysis disorder; however, the pathognomonic element of fibrinolysis, hypofibrinogenemia, was missing [15].

Although our patient received a single dose of low molecular weight heparin, the differential diagnosis with heparin-induced thombocytopenia, a platelet-activating anti-PF4-disorder, must be considered [16]:

- Classic heparin-induced thrombocytopenia (HIT), characterized by a drop in the number of platelets > 50% and/or thrombosis, occurring 5–10 days after initiation of treatment, was also considered. Patients develop anti-PF4 (platelet factor 4)-heparin antibodies, detectable by heparin enzyme immunoassay (EIA) and/or positive heparin-induced platelet activation test/HIPA assay [17,18]. We have performed a rapid immunological test to identify anti-PF4/heparin complex antibodies, which was negative; the T4 score was also calculated (and yielded the score 1), both findings making heparin-induced thrombocytopenia unlikely. High-specificity testing or functional evaluation of platelet activation were not performed. The use of the 4Ts clinical score in association with the rapid test for heparin-induced thrombocytopenia maintains its usefulness in clinical practice in the situation where it is not possible to perform tests such as EIA or HIPA assay.
- Autoimmune HIT (aHIT), in which the nature of anti-PF4 antibodies could be heparin-dependent or heparin-independent and includes four entities: delayed-onset HIT, persisting (refractory) HIT, heparin “flush” HIT, and fondaparinux-associated HIT. In all these entities, the PF4-dependent EIA test is positive, and the probability of thrombosis is >95% [16,19,20]. In our case, the patient did not receive non-heparin polyanionic pharmaceutical products capable of triggering HIT, nor fondaparinux.
- Post-knee arthroplasty spontaneous HIT characterized by symptomatic venous thrombosis and adrenal hemorrhage/necrosis [21].

Among other possible mechanisms responsible for postoperative thrombocytopenia, the following ones are worth taking into consideration:

- Hemodilution in association with increased consumption of platelets during surgical hemostasis: 30% of patients undergoing hip arthroplasty had a postoperative platelet count <150 × 10^9^/L [4,13]. These cases are physiological and not severe, with counts usually reaching no lower than 100 × 10^9^/L. However, our patient presented severe thrombocytopenia with sudden onset, which could not have been correlated with hemodilution only.
- Thrombocytopenia associated with splenomegaly (hypersplenism) could have been present before the surgical intervention. Immediate postoperative thrombocytopenia would be more severe in this situation, but in the present case, the CT scan showed normal spleen size (Figure 4) [13].
- In the etiology of postoperative hemorrhagic complications, the interaction between platelets and anesthetic agents has been considered, the responsible mechanisms being platelet dysfunction and alteration of platelet membrane receptors. Sevoflurane, halothane, and propofol inhibit platelet function in a reversible dose-dependent manner [22,23]. An antithrombotic effect was also observed in the case of locoregional anesthesia [24]. Isoflurane, enflurane, desflurane, barbiturates, etomidate, opioids, and muscle relaxants seem to have negligible effects on platelets, at therapeutic concentrations [22]. In the present case, this mechanism of thrombocytopenia was excluded, since the patient benefited from spinal anesthesia with bupivacaine.
- Post-transfusion thrombocytopenia (PTT—post-transfusion purpura). For notable blood loss that occurs as a result of major surgery, transfusion of packed erythrocytes, plasma products, and whole blood is often required. In some patients, mild to moderate thrombocytopenia develops after a transfusion of a large quantity of blood or blood products [25]. However, the mechanism standing behind this occurrence is represented by the formation of alloantibodies (following exposure to foreign antigens) and not autoantibodies (that react with self-antigens). Also, our patient required no blood products before being submitted to surgery and was not administered blood products in consequence, demonstrating that this mechanism cannot be incriminated [26].

Since all other causes of thrombocytopenia were excluded, we considered, in the present case, that the patient most likely developed a rare and severe form of postoperative thrombocytopenia through an immune mechanism. Immune-mediated postoperative thrombocytopenia (ITP) through the formation of circulating immune complexes is a cause of postoperative hemorrhagic complications and is characterized by severe thrombocytopenia (reaching counts of 1 × 10^9^/L) and immediate postoperative onset [1,27]. Therefore, this mechanism seemed the most accurate and feasible given our current medical context.

### 2.4. Therapeutic Management and Outcome

Daily local wound care and sterile dressing change were performed; antibiotic therapy continued postoperatively (vancomycin 2 g/day and ceftriaxone 2 g/day) along with transfusions with packed erythrocytes and platelet concentrates (ABO and RH system compatible), hemostatic agents, and antifibrinolytic agents: tranexamic acid (POD1-3). Methylprednisolone pulse therapy followed, 1 g/day for 3 days, with the persistence of severe thrombocytopenia and hemorrhagic syndrome (PLT 3 × 10^9^/L in POD3). We regarded this as corticosteroid refractoriness of the thrombocytopenia, thus intravenous immunoglobulin treatment was started with favorable initial evolution (PLT 15 × 10^9^/L in POD6 → 23 × 10^9^/L in POD12). Romiplostinum (thrombopoietin peptide mimetic) was administered in doses of 0.15 mcg (POD5) and 0.3 mcg (POD11); however, there was no persistence of the response, and a new mucosal hemorrhagic complication arose—gastrointestinal hemorrhage—with the decrease in platelet values to 2 × 10^9^/L in POD13.

**Figure 8:**
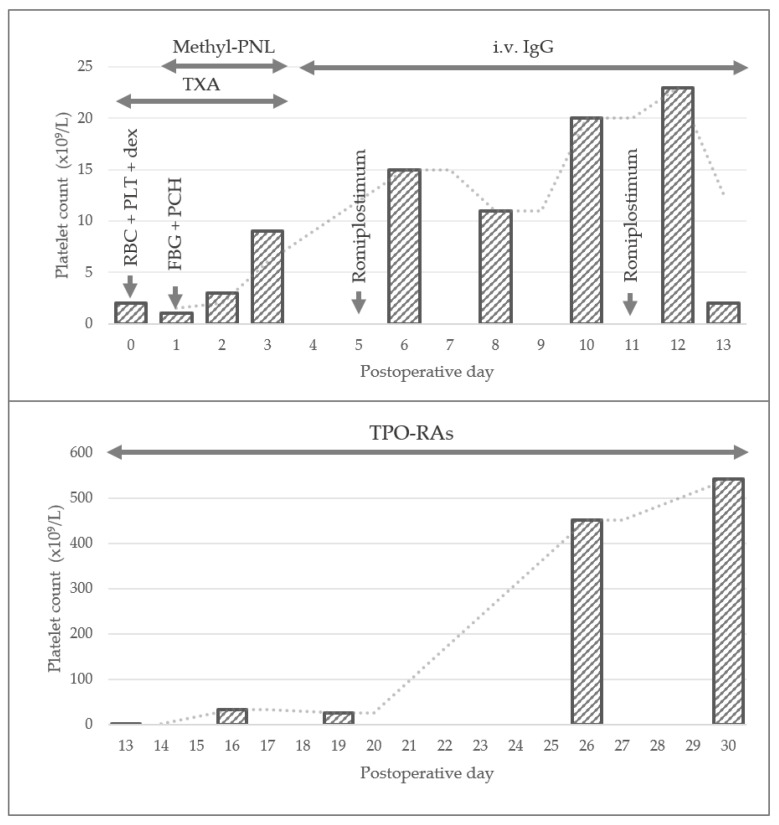
Evolution of platelet values in the first 30 days postoperatively under associated interventions. RBC = red blood cells; PLT = platelets; dex = desamethasone; FBG = Fibrinogen; PCH = Prothrombin Complex Human; TXA = Tranexamic Acid; Methyl-PNL = Methylprednisolone; i.v. IgG= Intravenous Human Normal Immunoglobulin G; TPO-RAs = Thrombopoietin Receptor Agonists.

On POD 14, the thrombopoietin receptor agonist—eltrombopag, 25 mg/day, was introduced, with the improvement of platelet values to 33 × 10^9^/L on POD 16 and 26 × 10^9^/L on POD 19, which prompted escalation to 50 mg/day. Shortly after, platelet counts recovered to the normal reference range (452 × 10^9^/L in POD 26 and 543 × 10^9^/L in POD 30), which required the interruption of treatment. There was favorable clinical evolution, marked by the disappearance of hemorrhagic syndrome stigmata and maintenance of platelets within normal limits (Figure 8).

## 3. Conclusions

The particularity of this case was that the postoperative thrombocytopenia was severe and life-threatening, refractory to multiple lines of treatment in contrast to the physiological postoperative thrombocytopenia, which is more frequent but mild, transitory, and no treatment is required.

Immune-mediated postoperative thrombocytopenia is a rare occurrence in clinical practice. However, in patients with severe postoperative thrombocytopenia (defined as platelet count nadir < 100 × 10^9^/L), alternative causes of thrombocytopenia (infection, drug-induced, immune) should be considered. While platelet-activating anti-PF4 disorders cannot be excluded in this case, it is well known that they strongly associate with thrombosis. A complex immune/non-immune mechanism might also be responsible for the condition reported in our case study.

Excessive postoperative bleeding due to thrombocytopenia can be life-threatening because it often appears on a background of hematologic anomalies, and occurs due to the surgery itself or because of additional agents used during the procedure. Clinical experience has shown that thrombocytopenia with early immediate postoperative onset is usually severe—grade 4, with platelet values lower than 10 × 10^9^/L; it is usually refractory to corticosteroids and substitution therapy; however, it quickly responds to treatment with thrombopoetin receptor agonists, an element that stands as proof and in favor of the immune mechanism behind it.

## Data Availability

The data that support the findings of this study are not openly available due to reasons of sensitivity and are available from the corresponding author upon reasonable request.
